# Reconstruction of medial meniscus posterior portion deficiency in pigs with an autologous patellar tendon graft: an experimental study

**DOI:** 10.1186/s13018-024-04684-1

**Published:** 2024-04-04

**Authors:** Zhian Chen, Anxu Li, Rongmao Shi, Ling Wang, Zijian Cao, Neng Mao, Zhihong Luo, Hongbo Tan

**Affiliations:** 1 Graduate School, Kunming Medical University, Kunming City, Yunnan Province China; 2 Department of Orthopaedics, People’s Liberation Army Joint Logistic Support Force 920th Hospital, Kunming City, Yunnan Province China

**Keywords:** Medial meniscus, Posterior root tear, Autologous tendon graft, Reconstruction of meniscus, Cartilage injury

## Abstract

**Objective:**

This study was performed to investigate the effectiveness of two surgical procedures, autologous patellar tendon graft reconstruction and trans-tibial plateau pull-out repair, using a pig model. The primary focus was to assess the repair capability of medial meniscus posterior portion (MMPP) deficiency, the overall structural integrity of the meniscus, and protection of the femoral and tibial cartilage between the two surgical groups. The overall aim was to provide experimental guidelines for clinical research using these findings.

**Methods:**

Twelve pigs were selected to establish a model of injury to the MMPP 10 mm from the insertion point of the tibial plateau. They were randomly divided into three groups of four animals each: reconstruction (autologous tendon graft reconstruction of the MMPP), pull-out repair (suture repair of the MMPP via a trans-tibial plateau bone tunnel), and control (use of a normal medial meniscus as the negative control). The animals were euthanized 12 weeks postoperatively for evaluation of the meniscus, assessment of tendon bone healing, and gross observation of knee joint cartilage. The tibial and femoral cartilage injuries were evaluated using the International Society for Cartilage Repair (ICRS) grade and Mankin score. Histological and immunohistochemical staining was conducted on the meniscus–tendon junction area, primary meniscus, and tendons. The Ishida score was used to evaluate the regenerated meniscus in the reconstruction group. Magnetic resonance imaging (MRI) was used to evaluate meniscal healing.

**Results:**

All 12 pigs recovered well after surgery; all incisions healed without infection, and no obvious complications occurred. Gross observation revealed superior results in the reconstruction and pull-out repair groups compared with the control group. In the tibial cartilage, the reconstruction group had ICRS grade I injury whereas the pull-out repair and control groups had ICRS grade II and III injury, respectively. The Mankin score was significantly different between the reconstruction and control groups; histological staining showed that the structure of the regenerated meniscus in the reconstruction group was similar to that of the original meniscus. Immunohistochemical staining showed that the degree of type I and II collagen staining was similar between the regenerated meniscus and the original meniscus in the reconstruction group. The Ishida score was not significantly different between the regenerated meniscus and the normal primary meniscus in the reconstruction group. MRI showed that the MMPP in the reconstruction and pull-out repair groups had fully healed, whereas that in the control group had not healed.

**Conclusion:**

Autologous patellar tendon graft reconstruction of the MMPP can generate a fibrocartilage-like regenerative meniscus. Both reconstruction and pull-out repair can preserve the structural integrity of the meniscus, promote healing of the MMPP, delay meniscal degeneration, and protect the knee cartilage.

## Introduction

The medial meniscus (MM) plays an important multifunctional role in maintaining the overall function of the knee joint, including transmission and distribution of the axial load between the tibiofemoral joints, shock absorption, joint stability and lubrication, proprioception, and nutrition supply [1–6]. The MM posterior portion (MMPP) bears a greater proportion of load through its strong bone attachment than does the anterior root [7, 8]; thus, it is easily injured. In arthroscopy, MMPP deficiency (MMPPD) accounts for 2.4% of all injuries, 10–21% of all meniscal tears, and 79.2% of all root tears [9–11]. MMPPD is divided into acute, subacute, and chronic injuries [12, 13], among which the main risk factors include severe varus deformity of the lower limbs, increasing age, female sex, higher body mass index, increased Kellgren–Lawrence grade, poor posture, meniscal compression, patellar softening, and bone and soft bone defects [14–19]. MMPPD is biomechanically equivalent to MM total resection in that it increases the contact pressure of the tibiofemoral joint, accelerates joint cartilage wear, gradually narrows the joint space, and ultimately progresses to medial compartment degenerative osteoarthritis [20]. Therefore, it is crucial to repair the MMPP and restore the biomechanical state of the joint.

Many treatment methods are available for MMPPD, including conservative management [15], meniscal debridement, partial meniscectomy, meniscal repair [21], and trans-tibial plateau pull-out repair [22]. Conservative management, meniscectomy, and suturing have poor efficacy [23, 24]. Trans-tibial plateau pull-out repair is the most commonly used surgical treatment, and 77.5% of patients with MMPPD undergo this procedure [9]. However, one study showed that 45% of MMPPs exhibited loose healing following pull-out repair, 36% exhibited scar healing, and 19% exhibited no healing [6]. Additionally, there are significant histological differences between the MMPP and the primary posterior root repaired through tibial pull-out [25]. Research on improving meniscal extrusion and knee osteoarthritis remains insufficient [26].

Animal studies have shown that the type I collagen (COL-I) fiber bundle of semitendinosus tendon tissue is identical to the meniscal portion, suggesting its potential for meniscal replacement [27]. The success of medial meniscal transplantation of the middle third of the patellar tendon of sheep and the subsequent clinical success of using the quadriceps tendon for medial meniscal transplantation suggests that a similar approach might be effective for the treatment of MMPPD [28]. Autologous tendon transplantation has been used for porcine acetabular labrum reconstruction. This approach can be used to completely or partially repair the labral defect and convert it into fibrocartilaginous tissue, implying that tendon tissue has similar plasticity to fibrocartilage [29]. The success of this procedure is mainly due to the ability of tendon-derived stem cells and synovial-derived mesenchymal stem cells to form fibrocartilage cells and synthesize proteoglycans, type II collagen (COL-II), and radial COL-I. There is no significant difference between the regenerated meniscus and the original meniscus [27, 30].

Therefore, reconstruction may be the most effective method for treating MMPPD. However, MMPPD reconstruction has not yet been tested in large animals. The present study was performed to compare the differences between autologous patellar tendon graft reconstruction and trans-tibial plateau pull-out repair using a pig model. We focused on the ability to repair the MMPPD, the overall structural integrity of the meniscus, and the ability to protect the femoral and tibial cartilage of the knee joints between the two surgical groups with the aim of providing experimental guidelines for clinical research.

## Materials and methods

### Animals

This study involved 12 healthy Yunnan small-eared pigs (9 months old, 18–22 kg). All animals were randomly selected to establish an acute injury model of the unilateral knee joint, with the MMPP positioned 10 mm away from the insertion point of the tibial plateau. The animals were divided into three groups: the reconstruction group (autologous tendon graft reconstruction of the MMPP, *n* = 4), pull-out repair group (MMPPD repaired by tibial plateau bone tunnel suture, *n* = 4), and control group (*n* = 4). The MM on one side of the knee joint did not undergo surgery and served as a negative control. The use of all animals adhered to the guidelines of the Ethics Review Committee of the 920th Hospital of the PLA Joint Logistics Support Force and was approved under Animal Protocol 920IEC/AF/61/2021 − 01.1.

### Establishment of MMPPD

**Fig. 1:**
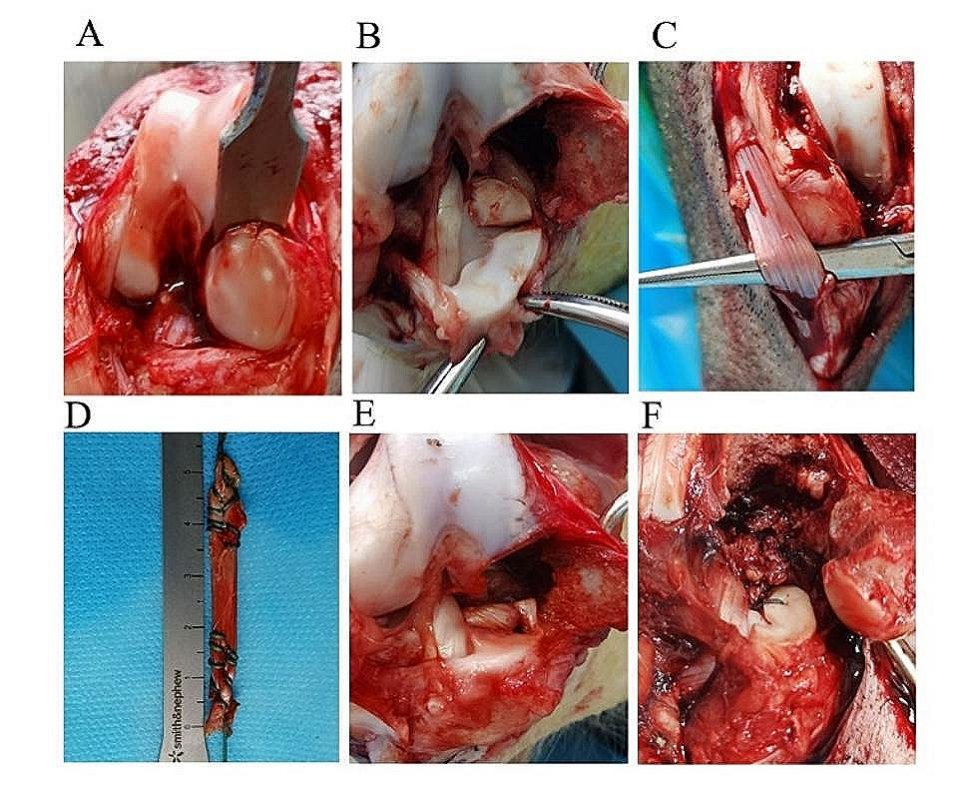
Surgical steps. (**A**) Medial femoral condyle osteotomy using an osteotome. (**B**) Creation of radial tear 10 mm from the MMPP root–tibial plateau projection. (**C**) Acquisition of patellar tendon. (**D**) Patellar tendon braid. (**E**) Braided tendon reconstruction of MMPP. (**F**) Pull-out repair

Modeling was performed according to the method described by Lee et al. [31] After induction of anesthesia by intravenous injection of 3% pentobarbital sodium at a dose of 1 mL/kg (Shandong Huamu Pharmaceutical Co., Ltd., China), the pigs were fixed on the operating table in the supine position. The skin was routinely prepared and sterilized. A skin incision was made over the knee joint using the conventional median approach; after reaching the patella, the medial patellar approach was used. The medial retinaculum and other tissues of the patella were opened. The patella was turned to a valgus position, and medial femoral condyle osteotomy was performed with an osteotome (Fig. 1A). After thorough exposure of the MMPP, a surgical blade was used to create a radial tear 10 mm from the insertion point of the MMPP on the tibial plateau (Fig. 1B). Because the patellar tendon is easier to obtain and its procurement inflicts minimal trauma to the animal model, half of the patellar tendon, measuring 5.5 cm in length (Fig. 1C), was used as the MMPP reconstruction graft in this experiment.

### Reconstruction group

After selecting the appropriate length of patellar tendon, the free end of the tendon was braided with a No. 2 ETHIBOND surgical suture (ETHICON, Raritan, NJ, USA) to create a 3-mm-diameter graft, and the braided tendon (Fig. 1D) was soaked in saline-diluted gentamicin solution. A 3-mm perforation was created on the meniscus at a distance of 5 mm from the stump of the MMPP, and the hole was dilated with a 3-mm-diameter Kirschner wire. The MM remnant on the tibial side, cartilage, and cortical bone were cleaned, and the cancellous bone was exposed. An anterior cruciate ligament positioning guide device (Shi Nehuis, USA) was aimed at the MMPP insertion. The angle was adjusted to 50° to 55°, and a 2-mm Kirschner wire was driven into the tibia (with the needle tip extending beyond the insertion point) for pre-positioning. A 6-mm-diameter tibial drill (Shi Nephew, USA) was then used for reaming (after the drill bit broke through the cortical bone, the electric drill was operated in reverse and moved back and forth several times to fully open the tunnel). The braided tendon was pulled into the treated meniscus by the shuttle thread method, and the tendon was then inserted into the bone tunnel by the same method. After resetting the femoral condyle, two 3.5-mm-diameter metal hollow screws were used for fixation. The reconstructed meniscus (Fig. 1E) was placed under a preload of 2 N [32], and a 2-mm-diameter Kirschner wire was used to puncture the bone marrow tract below the tibial tuberosity. Two sutures were fixed below the tibial tuberosity through the bone marrow tract [33].

### Pull-out repair group

The MMPP insertion stump, cartilage, and cortical bone were cleaned to expose the cancellous bone. The anterior cruciate ligament positioning guide device was aimed at the MMPP insertion, and the angle was adjusted to 50° to 55°. A single No. 2 ETHIBOND surgical suture was used to simply suture the end of the MM, and the suture was then pulled through the tibial tunnel by shuttling through the suture. The medial femoral condyle was repositioned and fixed with two 3.5-mm-diameter metal hollow screws. The repaired meniscus (Fig. 1F) was placed under a preload of 2 N [32], and a 2-mm-diameter Kirschner wire was used to puncture the bone marrow tract below the tibial tuberosity. Two sutures were fixed below the tibial tuberosity through the bone marrow tract [33].

### Control group

After cleaning the stump of the MMPP insertion point, no treatment was performed. The medial femoral condyle was reduced, and two 3.5-mm-diameter cannulated metal screws were used for fixation.

Finally, the knee joint cavity was irrigated with sterile saline, and the subcutaneous tissue and skin were closed using absorbable sutures. The pigs were returned to the animal center and allowed to bear weight and move freely with no activity restriction. Each pig was intramuscularly injected with sodium penicillin at 20,000 to 30,000 units/kg (Inner Mongolia Federal Animal Protection Pharmaceutical Co., Ltd., China) twice a day for 1 week to prevent postoperative infection.

### Gross observation

The pigs were euthanized by intravascular injection of pentobarbital (0.4 mL/kg) at 12 weeks postoperatively. Knee samples were collected from all pigs to examine the healing of the MMPP, assess the condition of the bone tunnel in the reconstruction group, grossly observe the tibial and femoral cartilage, and evaluate the specimens using the International Cartilage Repair Society (ICRS) grading system. Three joint surgeons who were blinded to the treatment methods scored all specimens individually, and the mean values were used for analysis.

### Histological staining

Target portions were separated from the collected fresh samples and fixed with 4% polyformaldehyde for 48 h. The femoral and tibial cartilage was decalcified with 10% ethylenediaminetetraacetic acid and 1% sodium hydroxide for 8 weeks and 5% formic acid for 2 weeks. The meniscus, patellar tendon, and decalcified cartilage were dehydrated through a graded alcohol series. They were then made transparent in xylene, immersed in paraffin wax (H22149; Leica Biosystems, Wetzlar, Germany), and embedded in paraffin. Sections were cut to 5-µm thickness and dried. They were then stained with hematoxylin–eosin (H3136, 230,251; Sigma-Aldrich, St. Louis, MO, USA) and safranin O with fast green (G1371; Solarbio, Beijing, China) and examined with a holographic scanning microscope. The sections were scanned to observe the changes in the intracellular and extracellular composition of the tissue. Three pathologists who were not involved in the experiment used the Ishida score [34] to quantitatively evaluate the MMPP in the reconstruction group and the Mankin score [35] to evaluate cartilage degeneration and injury. The Ishida score includes three aspects: the degree of binding between repaired regenerated tissue and native tissue, fibrochondrocytes, and safranin O staining. The Mankin score includes four aspects: cartilage results, cells, safranin O staining, and tidal line integrity.

### Immunohistochemical staining

The paraffin-embedded meniscal sections were placed in an oven at a constant temperature of 65 °C, deparaffinized, hydrated, permeabilized, rinsed with 0.01 mol/L phosphate-buffered saline (Tangier, China), subjected to antigen retrieval, rinsed again with 0.01 mol/L phosphate-buffered saline, blocked with 3% hydrogen peroxide, and incubated at room temperature for 30 min under protection from light. They were then blocked with 5% goat serum (ZLI-9021; Beijing Zhongshan Golden Bridge Biotechnology Co., Ltd., Beijing, China) and stored at 25 °C for 1 h. The appropriate concentration of primary antibody was added, and the sections were refrigerated at 4 °C overnight. PV9000 reagent (ZLI-9018; Beijing Zhongshan Golden Bridge Biotechnology Co., Ltd.) was added dropwise, and the sections were incubated at 37 °C for 30 min. PV9000 reagent (enzyme-labeled goat anti-mouse/rabbit) was then added dropwise, and the sections were incubated at 25℃ for 1 h. Diaminobenzidine was added for color development, the sections were allowed to sit for 5 to 10 min at room temperature, and distilled water was added to stop the color development. Hematoxylin counterstaining (in which the stained nucleus is blue) was performed, after which the sections were dried, made transparent, and sealed with neutral gum. The dried sections were scanned with a holographic scanning microscope to observe the changes in tissue COL-I and COL-II.

### Magnetic resonance imaging (MRI) examination

The intact right hind limb of each pig was removed. The knee joint was maintained in a straight position, and the specimen was placed in a 1.5T MRI machine (uMR 560; Shanghai United Imaging Healthcare Co., Ltd., Shanghai, China). With reference to Furumatsu et al. [36], the degree of MM extrusion (MME) and the ghost sign were described in the MRI coronal, sagittal, and axial views. The differences among the reconstruction group, pull-out repair group, and control group were evaluated and compared overall. Normal MRI was used as a negative control.

### Statistical analysis

All results are expressed as mean ± standard deviation. The Mankin score for the regenerated meniscus in the reconstruction group was analyzed by one-way analysis of variance, and the Ishida score was analyzed using a paired t-test (GraphPad Prism 8.0.1; GraphPad Software, San Diego, CA, USA). A P value of < 0.05 was considered statistically significant.

## Results

### Gross observation of meniscus

**Fig. 2:**
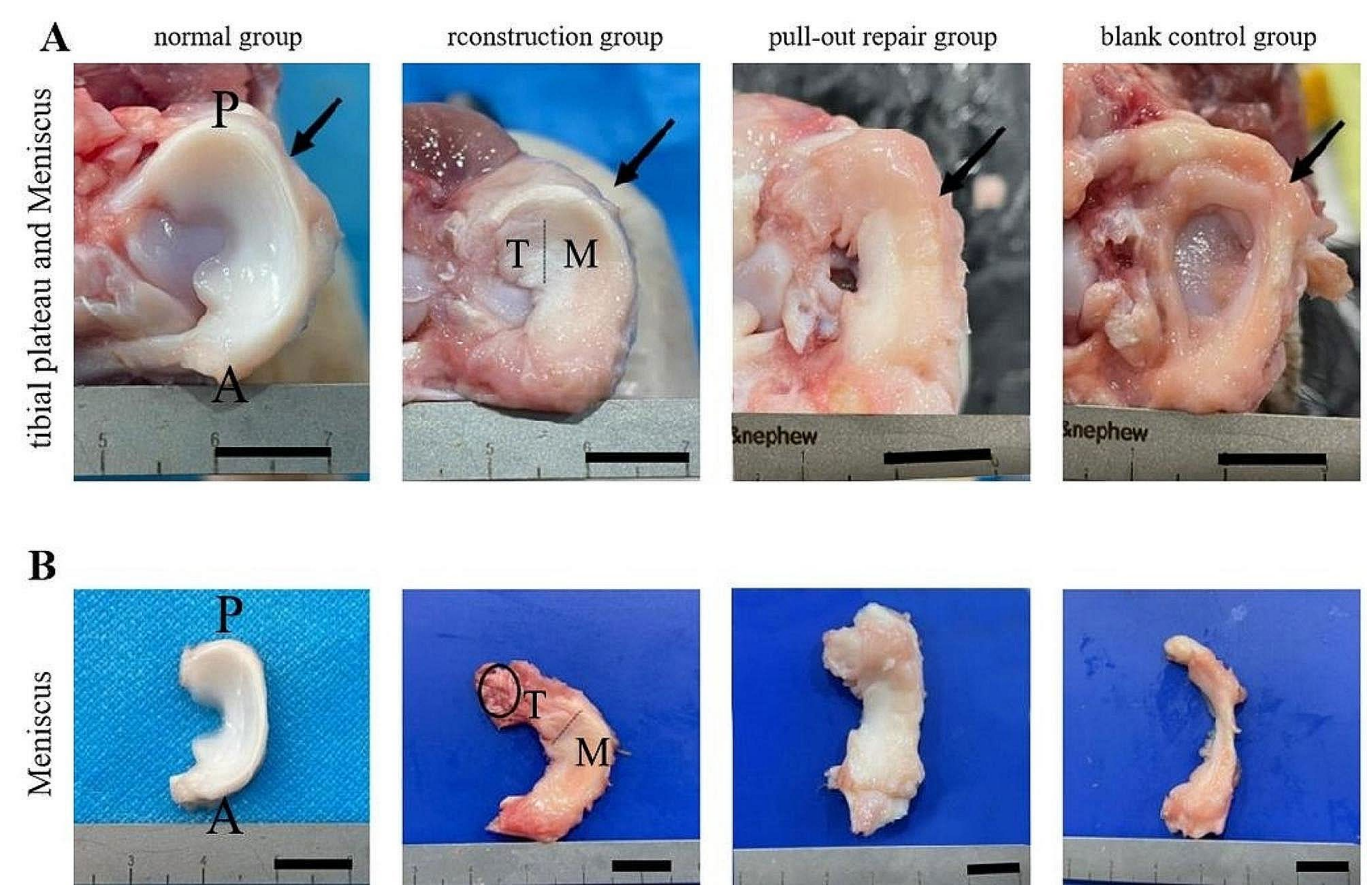
Gross observation of the meniscus. (**A**) Gross observation of the healing of the upper meniscus of the normal tibial plateau. (**B**) Gross observation of the meniscus. The black arrow indicates the medial tibial plateau. The black dotted line represents the healing junction between the regenerated meniscus and the native meniscus after autologous tendon graft reconstruction of the MMPP. The black oval represents the tendon that grew into the tibial tunnel and healed successfully. Scale bar = 1 cm. A, anterior; P, posterior; T, meniscus regenerated by tendon tissue; M, meniscus

At 12 weeks postoperatively, all pigs had recovered well, the incision had healed without infection, and no obvious complications had occurred. Gross observation in the normal group revealed that the meniscus was crescent-shaped and firmly growing on the tibial plateau without stenosis or atrophy. In the reconstruction group, the reconstructed zone of the MMPP had healed well and grown firmly on the tibial plateau, the MMPP was regenerated, and the overall meniscus was crescent-shaped and well-connected with the joint capsule without stenosis or atrophy. In the pull-out repair group, the MMPP had healed well and grown firmly on the tibial plateau. The whole meniscus was crescent-shaped and well-connected with the joint capsule. A large amount of hypertrophic scar tissue was seen in the MMPP. The meniscus in the control group showed atrophy, stenosis, and obvious scar tissue hyperplasia. This stenosis manifested as narrowing from the inner edge to the outer edge of the meniscus (Fig. 2A, B).

**Fig. 3:**
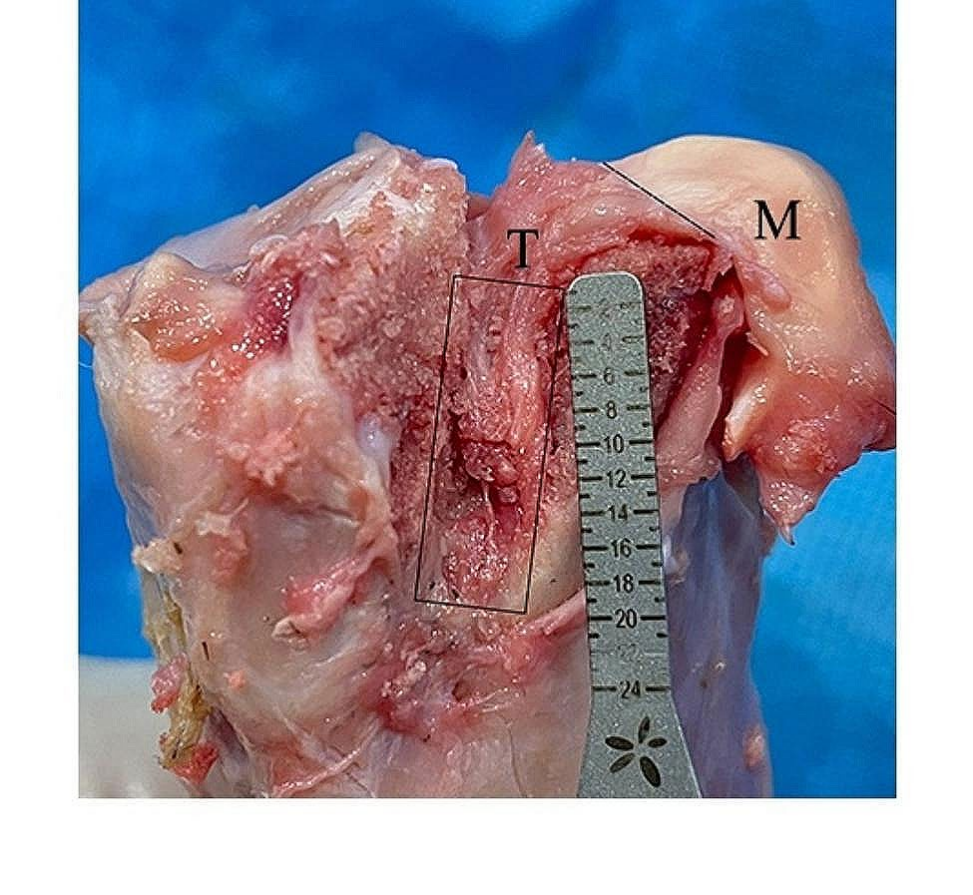
Tendon–bone healing. The black dashed line represents the healing junction between the regenerated meniscus and the native meniscus after autologous tendon graft reconstruction of the MMPP. The black box represents the tibial tunnel and the reconstructed tendon. T, meniscus regenerated by tendon tissue; M, meniscus

Gross examination in the reconstruction group revealed that the tendon had successfully grown into the bone tunnel (Fig. 3).

### Histological evaluation of meniscus

**Fig. 4:**
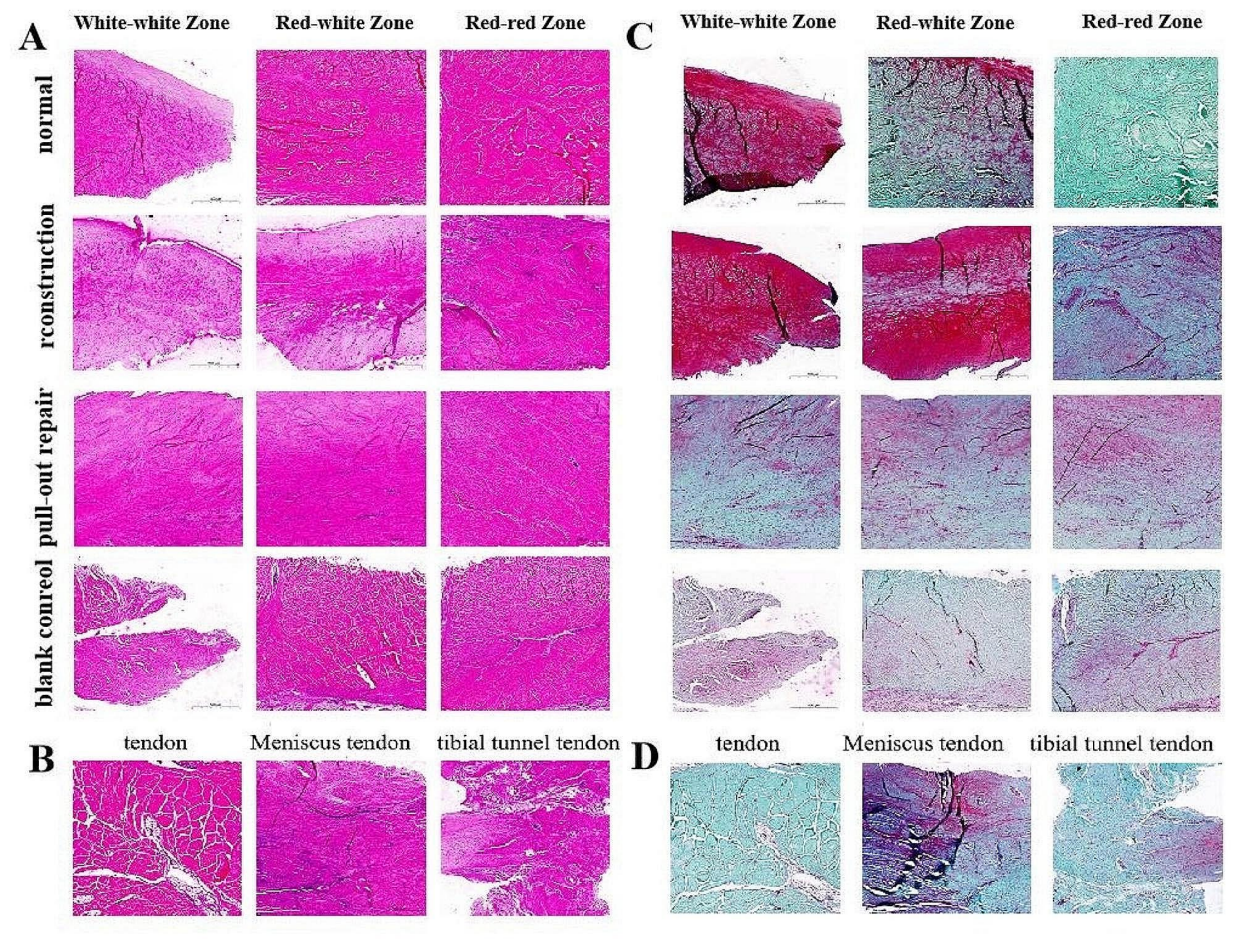
Regenerated meniscus and tibial tunnel tendon. **(A, B)** Hematoxylin–eosin staining. **(C, D)** Safranin O with fast green staining. Scale bar = 500 μm

Hematoxylin–eosin staining was performed to observe the histological changes in the MMPP (Fig. 4A, B). At 12 weeks after surgery, compared with the normal group, angiogenesis was observed in the red–red zone in the experimental group, with the reconstruction and pull-out repair groups showing histological changes similar to the normal meniscus in the white–white and red–white zones. In the control group, horizontal tears could be seen in the white–white zone; additionally, the white–white zone, red–white zone, and red–red zone of the meniscus contained equal amounts of uneven loose extracellular matrix and sparse fibrocartilage cell populations. The fibrocartilage cells, alone or in pairs, were located in clearly defined zones (Fig. 4A). Compared with the normal group, the reconstructed tendon graft was well connected with the meniscus, the extracellular matrix of the graft was uniform and dense, and the cells were composed of spindle-shaped fibroblasts and oval or round fibrocartilage cells. The extracellular matrix of the tendon in the tibial tunnel was uniform and loose, and the matrix density was increased compared with the normal tendon. The cells were composed of spindle-shaped fibroblasts and oval or round fibrocartilage cells, and the fibrocartilage cells outnumbered the fibroblasts (Fig. 4B).

The changes in MMPP protein polysaccharides were observed by safranin O with fast green staining (Fig. 4C, D). At 12 weeks postoperatively, the reconstructed meniscus showed strong protein polysaccharide staining in the white–white and red–white areas compared with the normal group, pull-out repair group, and control group, indicating protein polysaccharide synthesis in the fibrochondrocytes. Mild protein polysaccharide staining was observed in the red–red area of the meniscus, and this staining was more pronounced than in the pull-out repair and control groups. Similar slight protein polysaccharide staining was observed in the white–white, red–white, and red–red zones of the pull-out repair group and control group. The protein polysaccharide staining in the white–white and red–white zones of the two groups was not as obvious as that in the white–white and red–white zones of the normal meniscus (Fig. 4C). Compared with the normal group, the reconstruction group showed proteoglycan staining similar to that of the normal meniscus, indicating proteoglycan synthesis of fibrocartilage cells. Mild proteoglycan staining of the tendons in the tibial tunnel was observed (Fig. 4D).

**Fig. 5:**
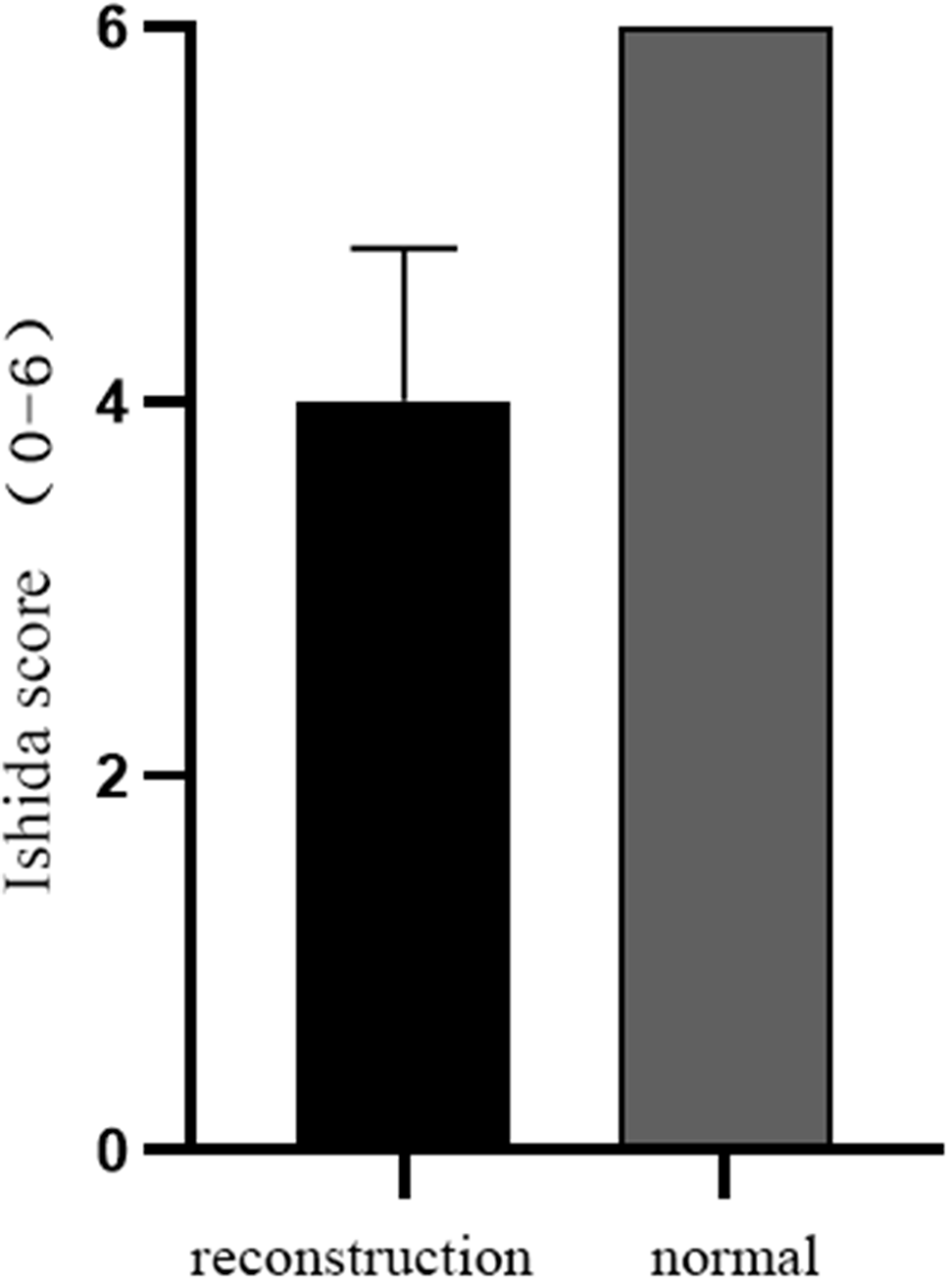
Ishida score of regenerated meniscal tissue. There was no significant difference in the Ishida score between the regenerated meniscal tissue in the reconstruction group and the normal meniscal tissue

The mean Ishida score of the regenerated meniscus in the reconstruction group was 4.00 ± 0.81, and the mean score in the normal group was 6.00 ± 0.00. There was no significant difference in the score between the regenerated meniscal tissue in the reconstruction group and the normal meniscal tissue (Fig. 5).

### Immunohistochemistry

**Fig. 6:**
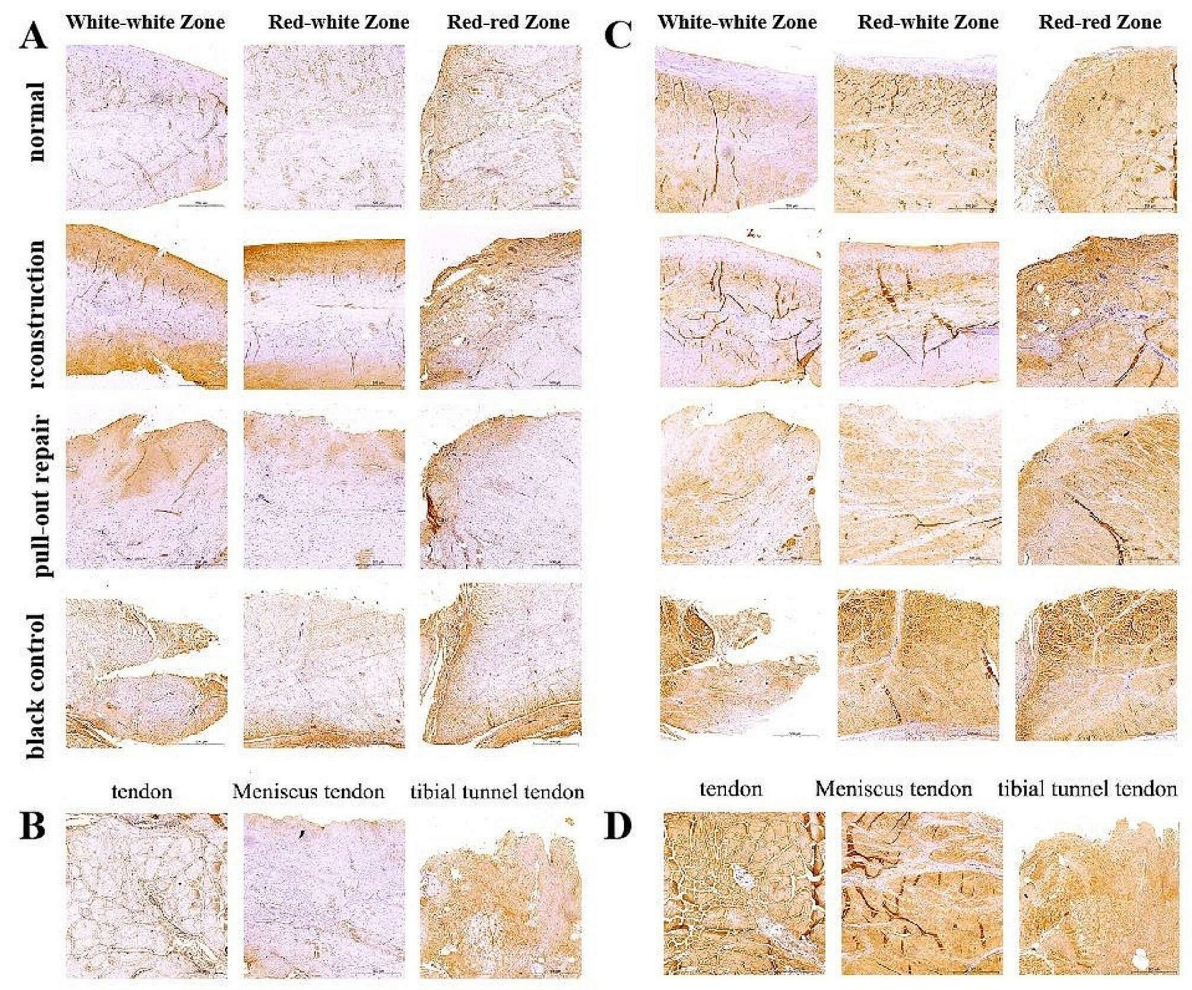
Regenerated meniscus and tibial tunnel tendon. **(A, B)** COL-I. **(C, D)** COL-II. Scale bar = 500 μm

Immunohistochemical staining was performed to observe the changes in COL-I (Fig. 6A, B) and COL-II (Fig. 6C, D) in the MMPP under different treatment conditions. At 12 weeks postoperatively, compared with the normal group, the white–white zones, red–white zones, and red–red zones of the MM in the reconstruction group showed moderate COL-I staining, mainly at the lateral border. Moderate COL-II staining was also present, but the red–red zones were more intensely stained. In the pull-out repair group, the white–white zones, red–white zones, and red–red zones of the MM showed the same COL-I staining results as in the reconstruction group. Moderate COL-II staining was also present, but the staining was relatively weak in the white–white zones. In the control group, the white–white zones, red–white zones, and outer red–red zones of the MM showed moderate COL-I staining, mainly at the lower edge of the meniscus. The staining was more obvious than in the reconstruction group and pull-out repair group. In addition, intense COL-II staining was observed. The normal tendon showed moderate COL-I and COL-II staining. At 12 weeks postoperatively, the reconstructed graft of the MMPP showed mild COL-I and COL-II staining, similar to that of the normal meniscus. The tendon in the tibial tunnel showed moderate COL-I and COL-II staining, but the degree of staining was stronger than that of the normal tendon.

### Assessment of articular cartilage

#### Gross observation

**Fig. 7:**
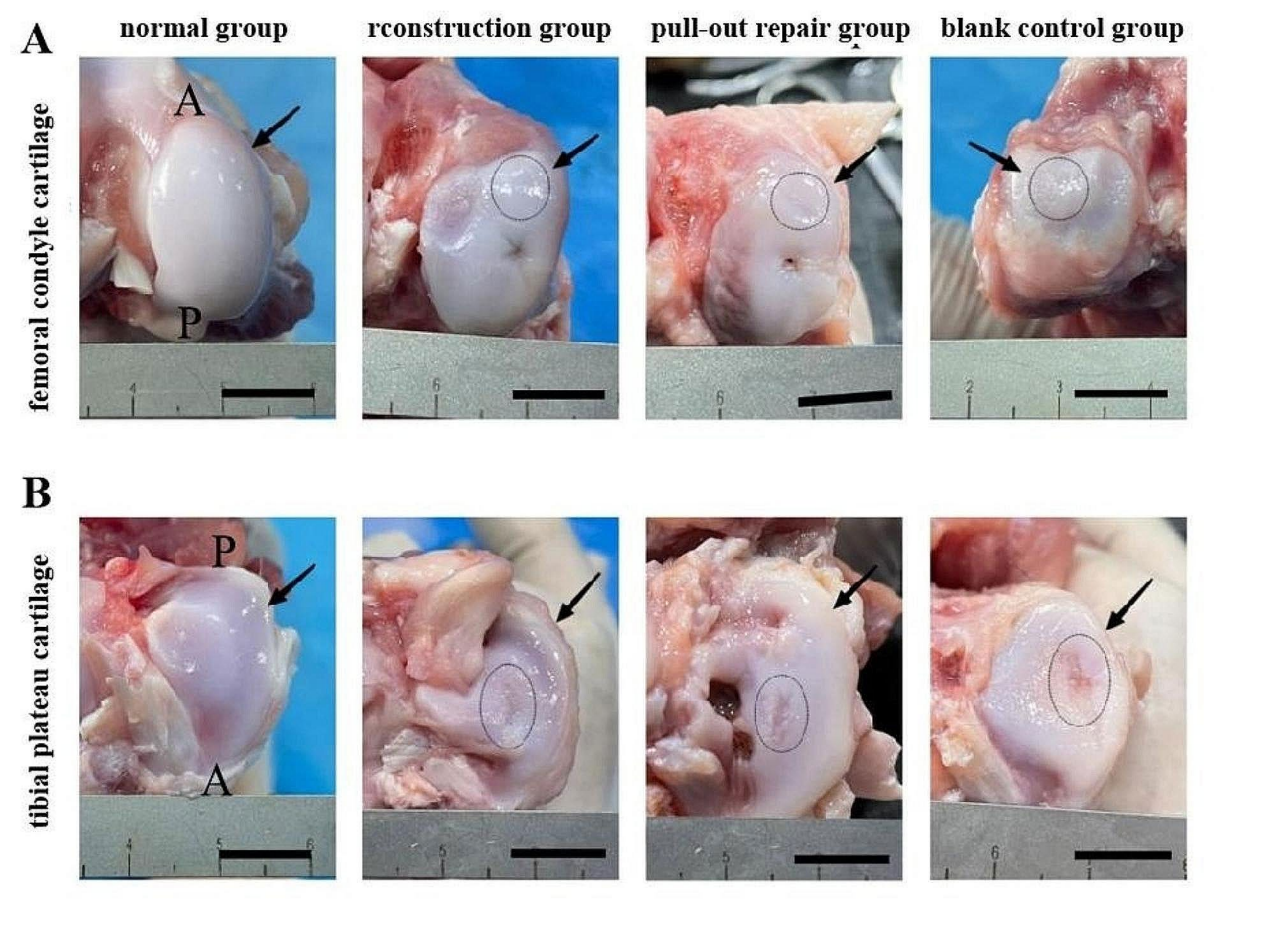
Gross observation of normal femoral condyle and tibial plateau versus femoral condyle and tibial plateau in reconstruction group, pull-out repair group, and control group at 12 weeks postoperatively. (**A**) Femoral condyle. The black arrow indicates the medial condyle of the femur, and the black circle indicates cartilage damage in the weight-bearing area of the femoral condyle. (**B**) Tibial plateau. The black arrow indicates the medial tibial plateau, and the black ellipse indicates cartilage damage in the weight-bearing area of the tibial plateau. Scale bar = 1 cm. A, before; P, after

Compared with the normal femoral condyle cartilage, the weight-bearing area of the femoral condyle cartilage in the reconstruction group, pull-out repair group, and control group was slightly worn at 12 weeks postoperatively and exhibited ICRS grade I injury (Fig. 7A). In the reconstruction group, the weight-bearing area of the tibial plateau cartilage was slightly worn, showing ICRS grade II injury. In the pull-out repair group, the cartilage-bearing area of the tibial plateau was moderately worn and exhibited ICRS grade II injury. In the control group, the weight-bearing area of the tibial plateau cartilage was severely worn with ICRS grade III injury (Fig. 7B).

### Histological evaluation of cartilage

**Fig. 8:**
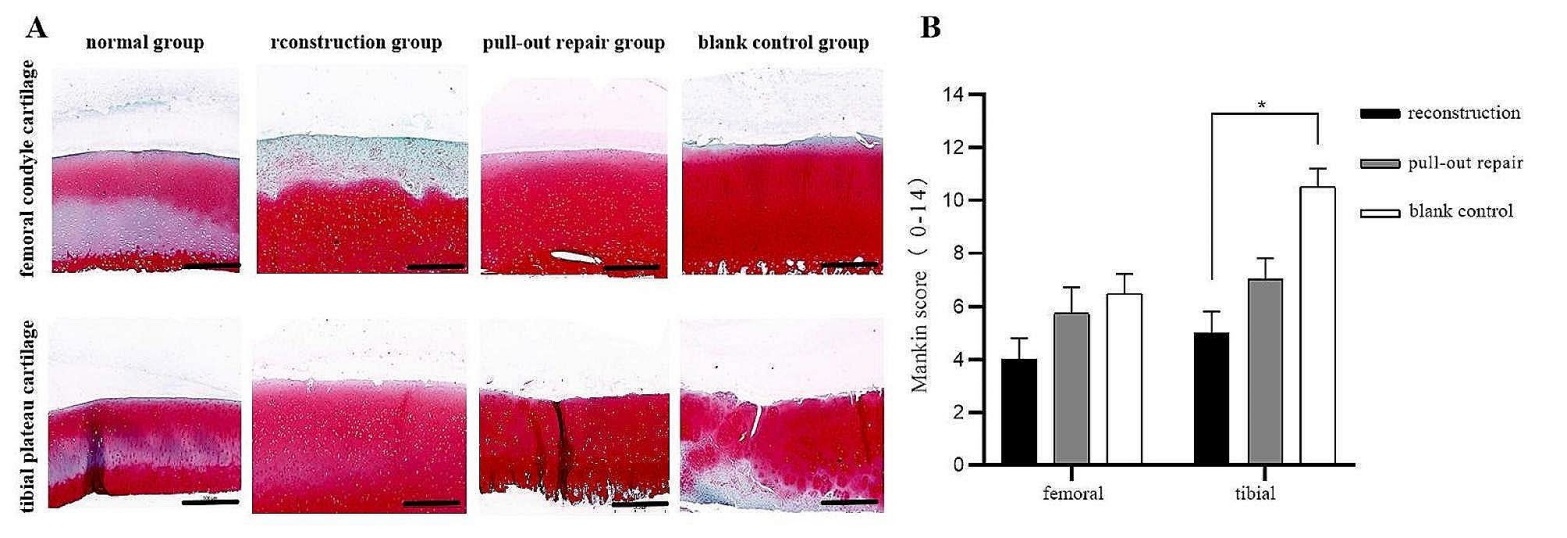
Safranin O with fast green staining of weight-bearing area of medial femoral condyle and medial tibial plateau cartilage. (**A**) Mankin score of medial femoral condyle. (**B**) Mankin score of medial tibial plateau cartilage. Scale bar = 500 μm. *Significant difference (*P* < 0.05)

The results of safranin O with fast green staining were similar to the macroscopic results. Compared with the pull-out group and control group, the reconstruction group showed less damage to the weight-bearing area of the femoral condyle cartilage and tibial plateau cartilage at 12 weeks postoperatively (Fig. 8A). The Mankin score of the femoral cartilage showed no significant difference among the three groups. However, the Mankin score of the tibial cartilage was significantly different between the reconstruction group and control group (*P* < 0.05) (Fig. 8B).

### MRI examination

**Fig. 9:**
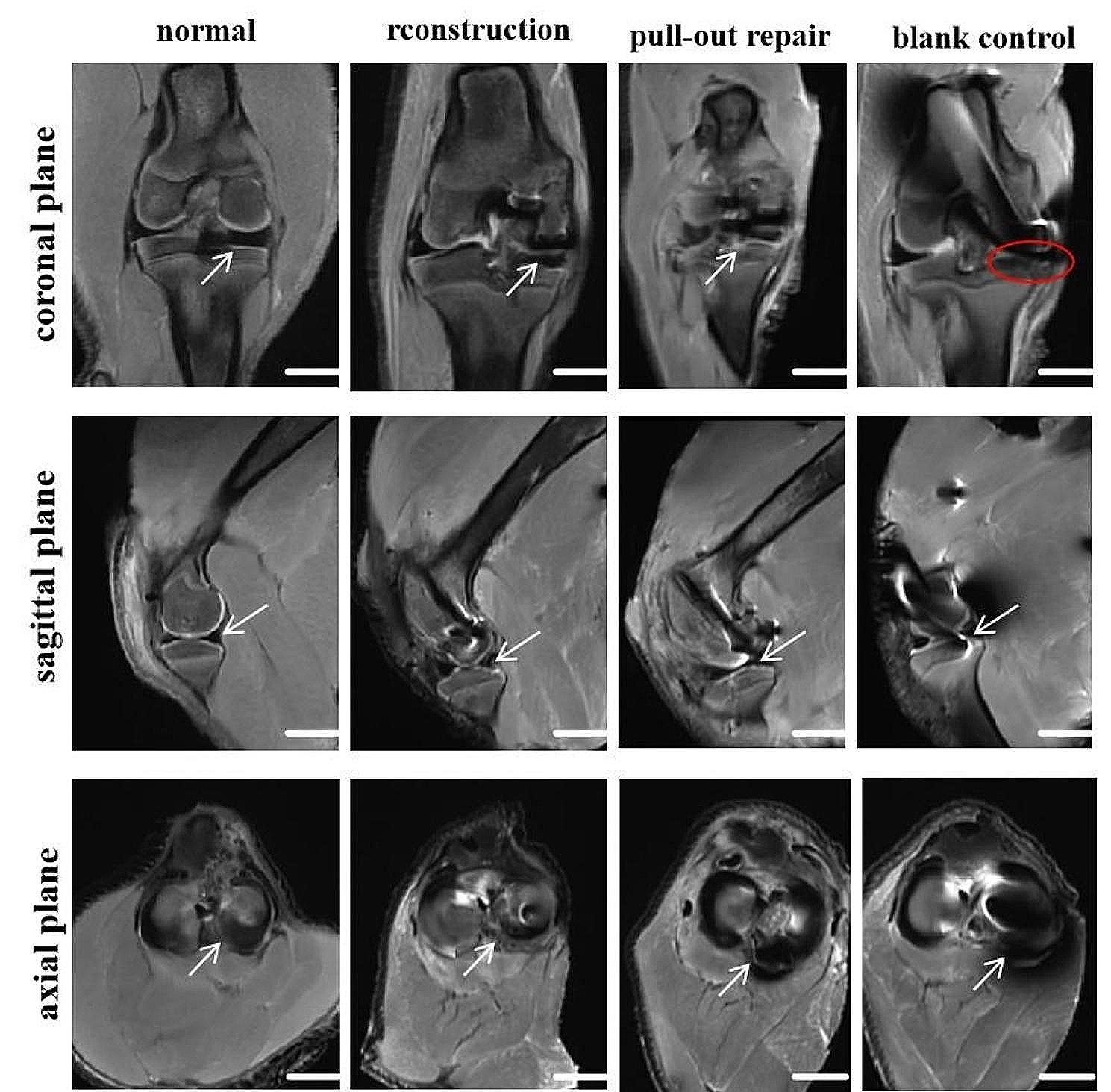
Coronal, sagittal, and axial MRI. White arrows indicate the MMPP. Red circles indicate complete extrusion and disappearance of the MM. Scale bar = 1 cm

At 12 weeks postoperatively, the MME, giraffe neck sign, and longitudinal fissure sign in each group were observed on the coronal MRI scan; the ghost sign was observed on the sagittal scan; and the radial tear sign was observed on the axial scan (Fig. 9). MRI showed that the MMPP in the reconstruction group had completely healed, the meniscus was stably fixed on the tibial plateau, and there was no indication of MME, the giraffe neck sign, the longitudinal fissure sign, the ghost sign, or the radial tear sign. The MMPP in the pull-out repair group had completely healed, and the meniscus was stably fixed on the tibial plateau. However, there was slight extrusion toward the medial side. There was no giraffe neck sign, longitudinal fissure sign, ghost sign, or radial tear sign. In the control group, the MM was completely extruded and no posterior root healing was evident.

## Discussion

The primary finding of the present study is that autologous tendon graft reconstruction of the MMPP promoted its healing, maintained the overall structural integrity of the meniscus, and delayed degenerative changes in the tissue. In addition, menisci that had undergone autologous tendon graft reconstruction were similar to the normal menisci with respect to the tibial plateau attachment area, cell type distribution, collagen type, and proteoglycan content. The protective effects of pull-out repair on the femoral condyle and tibial plateau cartilage of the knee joint were similar to those achieved in the MMPP reconstruction group, but the latter technique had advantages with respect to meniscus and cartilage protection. Thus, autologous half-patellar tendon reconstruction of the MMPP is a promising approach that provides a clinically feasible treatment option for MMPPD.

Compared with meniscal allografts and synthetic scaffolds, autologous tendon grafts are not at risk of autoimmune rejection, are not associated with a risk of disease transmission, and have relatively high biocompatibility and stability. In their previous study, Kohn et al. [28] removed the MM from Merino sheep, used the middle third of the patellar tendon to reconstruct the meniscus, and found that meniscal reconstruction had a protective effect on the femoral condyle and tibial plateau cartilage. Shi et al. [29] used an autologous gluteus medius tendon graft to unilaterally reconstruct the acetabular labrum, and all grafts eventually joined the acetabulum during healing and transformed into an acetabular labrum. This increased the depth of the acetabular fossa, further stabilized the hip joint, and reduced wear of the articular cartilage. In addition, Li et al. [27] considered that the graft density and scaffold structure affect tissue remodeling and regeneration. They performed successful regenerative meniscus replacement using fresh semitendinosus tendon grafts in rabbits, achieving fibrocartilage-like properties and cartilage protection.

In the present study, we used an autologous half-patellar tendon as an MMPP reconstruction graft. We chose this technique because compared with hamstring muscle transfer, patellar tendon reconstruction of the anterior cruciate ligament can be associated with earlier motor recovery [37]. We found that the autologous patellar tendon graft had transformed into a regenerated meniscus 12 weeks following surgery and protected the femoral condyle and tibial plateau. Gross inspection revealed only slight wear and little difference in the femoral condyle cartilage among the reconstruction, pull-out repair, and control groups. Although there were no significant differences among the groups, the Mankin score tended to be better in the reconstruction group than in the pull-out repair group, and the control group had the worst score. However, gross and histological inspection of the tibial plateau cartilage revealed clear differences among the groups. There was a significant difference in the Mankin score between the reconstruction and control groups, but the reconstruction group tended to have a better score than the pull-out repair group. The control group still had the worst score. Thus, as long as the tension of the meniscal hoop can be restored after MMPPD, the articular cartilage can be protected. This may be achieved by either pull-out repair or reconstruction.

The meniscus contains numerous cellular components. Angiogenic and fibroblast-like cells are mainly present in the red–red and red–white zones, which are rich in COL-I and sulfated glycosaminoglycans. By contrast, the white–white zones are not vascularized and principally contain hyaline chondrocyte, COL-II, and sulfated glycosaminoglycans [37–39]. Twelve weeks after surgery in the present study, both the number of cells in the graft and the density of the extracellular matrix had increased, which mediated remodeling of the graft. The number of cells in the regenerated meniscus was higher than that in the normal meniscus, the fibroblasts outnumbered the fibrochondrocytes, and the amount of proteoglycan was similar to that in the normal meniscus. In addition, the remodeling of COL-I and COL-II fibers in the regenerated meniscus had made them similar to those in the normal meniscus. These findings suggest that the regenerated meniscus formed by MMPP reconstruction with an autologous tendon graft can substitute for a normal meniscus.

In the present study, MRI scans of the porcine knee joints were performed 12 weeks after surgery. When MMPPD occurs, the MM is extruded toward the medial border of the tibial plateau as the tibiofemoral joint is squeezed. MME is clinically defined as significant medial displacement of the MM relative to the central edge of the medial tibial plateau [40], and whether this extrusion exceeds 3 mm is an important index of its severity [41, 42]. However, there is currently no uniform definition or standard for the assessment of porcine MME. The coronal, sagittal, and axial MRI scans performed in the present study showed no MME in the reconstruction group, as for the normal meniscus. However, the pull-out repair group showed slight medial MME and a mild ghost sign. In addition, the control group showed complete MME and no posterior root healing. Thus, either autologous tendon graft reconstruction or pull-out repair for the treatment of MMPPD can ensure healing of the MMPP and reduce medial MME; however, MMPP reconstruction is more effective because it preserves the overall hoop tension of the meniscus and protects the joint.

In conclusion, autologous patellar tendon graft reconstruction of the MMPP can generate a fibrocartilage-like regenerative meniscus, and both reconstruction and pull-out repair can preserve the structural integrity of the meniscus, promote healing of the MMPP, delay degeneration of the meniscus, and protect the knee cartilage. These findings are expected to provide guidance for future clinical research of these techniques.

## Data Availability

All datasets presented in this study are included in the article.
